# Comprehensive analysis of immunoglobulin and clinical variables identifies functional linkages and diagnostic indicators associated with Behcet’s disease patients receiving immunomodulatory treatment

**DOI:** 10.1186/s12865-021-00403-1

**Published:** 2021-02-22

**Authors:** Linlin Cheng, Yang Li, Ziyan Wu, Liubing Li, Chenxi Liu, Jianhua Liu, Jiayu Dai, Wenjie Zheng, Fengchun Zhang, Liujun Tang, Xiaobo Yu, Yongzhe Li

**Affiliations:** 1 Department of Clinical Laboratory, Peking Union Medical College Hospital, Peking Union Medical College and Chinese Academy of Medical Sciences, No.1 Shuaifuyuan Wangfujing Dongcheng District, Beijing, 100730 China; 2 State Key Laboratory of Proteomics, Beijing Proteome Research Center, National Center for Protein Sciences (Beijing), Beijing Institute of Lifeomics, No. 38, Life Science Park Road Changping District, Beijing, 102206 China; 3 Department of Rheumatology and Clinical Immunology, Peking Union Medical College Hospital, Peking Union Medical College and Chinese Academy of Medical Sciences, Key Laboratory of Rheumatology and Clinical Immunology, Ministry of Education, Beijing, 100730 China; 4 Department of Laboratory Medicine, Shengjing Hospital of China Medical University, Shenyang, 110004 China

**Keywords:** Corticosteroids, Immunosuppressants, Immunoglobulin, Plasma microarray, Clinical variable

## Abstract

**Background:**

Behcet’s disease (BD) is a relapsing systemic vascular autoimmune/inflammatory disease. Despite much effort to investigate BD, there are virtually no unique laboratory markers identified to help in the diagnosis of BD, and the pathogenesis is largely unknown. The aim of this work is to explore interactions between different clinical variables by correlation analysis to determine associations between the functional linkages of different paired variables and potential diagnostic biomarkers of BD.

**Methods:**

We measured the immunoglobulin proteome (IgG, IgG1–4, IgA, IgA1–2) and 29 clinical variables in 66 healthy controls and 63 patients with BD. We performed a comprehensive clinical variable linkage analysis and defined the physiological, pathological and pharmacological linkages based on the correlations of all variables in healthy controls and BD patients without and with immunomodulatory therapy. We further calculated relative changes between variables derived from comprehensive linkage analysis for better indications in the clinic. The potential indicators were validated in a validation set with 76 patients with BD, 30 healthy controls, 18 patients with Takayasu arteritis and 18 patients with ANCA-associated vasculitis.

**Results:**

In this study, the variables identified were found to act in synergy rather than alone in BD patients under physiological, pathological and pharmacological conditions. Immunity and inflammation can be suppressed by corticosteroids and immunosuppressants, and integrative analysis of granulocytes, platelets and related variables is likely to provide a more comprehensive understanding of disease activity, thrombotic potential and ultimately potential tissue damage. We determined that total protein/mean corpuscular hemoglobin and total protein/mean corpuscular hemoglobin levels, total protein/mean corpuscular volume, and plateletcrit/monocyte counts were significantly increased in BD compared with controls (*P* < 0.05, in both the discovery and validation sets), which helped in distinguishing BD patients from healthy and vasculitis controls. Chronic anemia in BD combined with increased total protein contributed to higher levels of these biomarkers, and the interactions between platelets and monocytes may be linked to vascular involvement.

**Conclusions:**

All these results demonstrate the utility of our approach in elucidating the pathogenesis and in identifying novel biomarkers for autoimmune diseases in the future.

**Supplementary Information:**

The online version contains supplementary material available at 10.1186/s12865-021-00403-1.

## Introduction

Behcet’s disease (BD) is a chronic and relapsing vascular autoimmune/autoinflammatory disease of unknown cause, displaying involvement of multiple organs [1, 2]. BD is highly prevalent in countries along the “Silk Road”; it can be induced by persistent and excessive immune reactions via autoantigen-activated dendritic cells and T or B cells and leads to endothelial cell damage and vasculitis [3]. Increases in human IgM, IgG and IgA levels have been found in BD patients, and the production of immunoglobulin isotypes is associated with mucocutaneous, ocular and systemic involvement in naïve active BD patients [4, 5].

Clinically, corticosteroids and immunosuppressants are employed in rheumatoid immune diseases to attenuate the inflammatory response and tissue damage and relieve clinical symptoms due to their anti-inflammatory and immunosuppressive effects on the immune system [4, 6–9]. However, their long-term use may lead to immune disorders [4, 7] and increased susceptibility to viral infection [10]. For example, Djaballah-Ider et al. found that corticosteroid therapy significantly reduced serum immunoglobulin isotype markers [4] and inflammatory mediators related to disease pathogenesis, including IL-18 and IFN-γ regardless of the clinical manifestations in BD [7]. Direskeneli et al. found that thalidomide has both anti-inflammatory and regulatory effects in BD, decreasing the levels of the TNF-α receptor, CD8/CD11b + T cells and natural killer cells in early treatment while increasing CD4 + CD45RO+ memory T and γδ + T cells in later treatment [9].

The systems in the human body (e.g., coagulation, inflammation, etc.) are known to execute their functions cooperatively instead of alone, which can be reflected by clinically measured variables [11–15]. For example, in a study of 48 participants with cirrhosis and nonalcoholic fatty liver disease, Niu et al. revealed the association of five plasma proteins (DPP4, ANPEP, TGFBI, PIGR and APOE) with liver enzymes through a global correlation map of clinical and proteomic data, implying their associations with cirrhosis and nonalcoholic fatty liver disease [12]. Nathan et al. investigated the personal, dense and dynamic data from 108 individuals during a 9-month period and generated a correlation network between clinical variables, proteomes and genome sequences, which revealed communities of related analytes associated with physiology and disease. For example, the negative correlation between levels of cystine in plasma and polygenic risk scores for inflammatory bowel disease revealed that genetic predisposition of diseases may be manifested by analyte changes and suggested that supplementation with cystine in a healthy population at high risk may stop the transition to disease by preventing inflammation and oxidative damage. Nathan proposed that measurement of personal data clouds over time can improve understanding of health and disease and are the essence of precision medicine.

Evidence has suggested that multiple pathological pathways are involved in BD with no single common denominator related with BD [2]. However, no common or dominant pathological factor for BD has been identified until now. Moreover, the correlations of clinical variables in BD and their associations with BD diagnosis, progression and therapy are largely unknown. The hypothesis of this work is to explore the interactions between different clinical variables by correlation analysis to determine the associations between the functional linkages of different paired variables and potential diagnostic biomarkers of BD. To address this issue, we first measured the immunoglobulin proteome (IgG, IgG1–4, IgA, IgA1–2) using a plasma microarray and performed a comprehensive correlation analysis of the immunoglobulin proteome and 29 clinical variables. We defined the physiological, pathological and pharmaceutical relationships based on the correlations of all variables in the healthy controls (HCs) and BD patients without and with immunomodulatory therapy. Furthermore, we calculated the ratio changes between clinical variables to identify the specific indicators for the diagnosis of BD and differential diagnosis from other types of vasculitis.

## Materials and methods

### Demographic and clinical characteristics of subjects

All plasma samples were obtained from the Peking Union Medical College Hospital (Table 1), where BD patients were diagnosed according to the 1990 International Study Group (ISG) criteria [16] and the International Criteria for Behcet’s Disease (ICBD) [17], and patients with Takayasu arteritis (TA) and those with ANCA-associated vasculitis (AAV) were diagnosed respectively according to [18, 19].Furthermore, all patients with BD were assigned to four groups according to medication use, which included BD patients without treatment (BD-N), treatment with corticosteroids (BD-C), treatment with immunosuppressants (BD-I) or treatment with both (BD-C&I). Patients using immunosuppressants are defined as those who are under drug treatments, including Azathioprine, Cyclosporine, Thalidomide, Cyclophosphamide, Leflunomide, Hydroxychloroquine and Tripterygium glycosides. Blood samples were anticoagulated with EDTA, centrifuged at 12,000 rpm for 10 min, and the upper plasma layer was collected and frozen at − 80 °C until use. This study was approved by the Medical Ethics Committee of Peking Union Medical College Hospital (JS-2049), informed consent was obtained from all subjects. All research on humans was performed in accordance to the Declaration of Helsinki.

**Table 1:** Demographic and clinical characteristics of subjects

		BD-N	BD-C	BD-I	BD-C&I	HC	AAV	TA
	N	15	12	8	28	66	/	/
**Discovery set**	Age(y)	35.40 ± 10.16	27.25 ± 13.18	30.50 ± 6.80	31.04 ± 9.800	49.17 ± 13.51	**/**	**/**
	Sex(M/F)	10/5	7/5	4/4	20/8	31/35	**/**	**/**
	ICBD	5.73 ± 2.63	5.75 ± 1.36	5.00 ± 1.53	6.643 ± 1.890	/	**/**	**/**
**Validation set**	N	27	5	16	/	30	**18**	**18**
	Age(y)	37.81 ± 11.60	34.20 ± 12.40	36.94 ± 13.69	/	38.40 ± 11.40	**51.89 ± 14.07**	**33.94 ± 10.46**
	Sex(M/F)	8/19	3/2	5/11	/	14/16	**7/11**	**7/11**
	ICBD	5.19 ± 1.62	6.60 ± 0.89	5.50 ± 1.32	/	/	**/**	**/**

*BD-N* BD patients not receiving corticosteroid or immunosuppressant treatment, *BD-C* BD patients receiving only corticosteroid treatment, *BD-I* BD patients receiving only immunosuppressant treatment, *BD-C&I* BD patients receiving both corticosteroid and immunosuppressant treatment

### Quantification of the immunoglobulin proteome using plasma microarray

All plasma samples were retrieved from the − 80 °C freezer, thawed on ice and centrifuged at 12,000 rpm for 10 min at 4 °C. Five microliters of each plasma sample was diluted with 0.02% BSA (phosphate-buffered saline, PBS, pH = 7.4) according to the immunoglobulin subtype (IgG2 IgG3, IgG4 and IgA2:10×; IgA1: 100×; IgG, IgA, IgG1: 500×). Standard immunoglobulin proteins were obtained commercially, including IgG (ZSGB-BIO, Beijing, China), IgA (Bersee Technology Co. Ltd., Beijing, China), IgG1 (Sino Biological Inc., Beijing, China), IgG2 (Sino Biological Inc.), IgG3 (Sino Biological Inc.), IgG4 (Sino Biological Inc.), IgA1 (Fitzgerald Industries International, Massachusetts, USA) and IgA2 (Fitzgerald Industries International). BSA (1 mg/ml) and 1x PBS (pH 7.4) were used as blank controls. All the samples were printed on the modified slide surface (CapitalBio Technology Co., Ltd., Beijing, China) in duplicate by a Smart-Arrayer™ 136 microarrayer (CapitalBio Technology Co., Ltd., Beijing, China).

Prior to the assay, the plasma microarray was first blocked with 1% BSA at room temperature for 1 h. The detection of immunoglobulin proteins in plasma was performed by incubation for 30 min with the appropriate fluorescein-labeled detection antibodies, including Donkey anti-hIgG(Fc) Alexa Fluor 555 and Rabbit anti-hIgA(Fc) Alexa Fluor 647 (Jackson Immuno Research, Pennsylvania, USA), Mouse Anti-Human IgG1 Hinge-Alexa Fluor® 488, Mouse Anti-Human IgG2 Fc-Alexa Fluor® 488, Mouse Anti-Human IgG3 Hinge-Alexa Fluor® 647, Mouse Anti-Human IgG4 Fc-Alexa Fluor® 647, Mouse Anti-Human IgA1-Alexa Fluor® 647 and Mouse Anti-Human IgA2-Alexa Fluor® 488 (SouthernBiotech, Birmingham, USA). The unbound molecules were removed by washing the slide with 0.05% PBST three times and deionized water two times in the dark. Then, the resulting slide was air-dried and scanned by GenePix® 4300A (Molecular Devices, California, USA) at a wavelength of 488 nm (IgG1, IgG2 and IgA2), 532 nm (IgG) or 635 nm (IgA, IgA1, IgG3 and IgG4).

The quantification of immunoglobulin proteins in plasma was performed by using a standard curve fitted with a 4- or 5-parameter logistic model using the “nplr package” in R as previously described [20].

### Measurement of clinical variables

All plasma samples were removed from the − 80 °C freezer, thawed on ice and centrifuged at 12,000 rpm for 10 min at 4 °C. The basic and clinical information of patients was obtained from the Hospital Information System of Peking Union Medical College Hospital, including age, sex, disease history, clinical symptoms and clinical treatment information with corticosteroids and/or immunosuppressants (Table 1). The results of the laboratory tests at the time of sample collection were obtained from the Laboratory Information Management System, including clinical chemistry, clinical immunology, hematology, etc. The abbreviations and full names of all clinical variables are shown in Table S1. Routine blood tests were completed by a Siemens ADVIA2120 or Sysmex XN9100 analyzer (Siemens, Munich, Germany; Sysmex America, Illinois, USA); ESR tests were completed by a Greiner MONITOR-S analyzer (Greiner Bio-one GmbH, Kremsmünster, Austria); CRP tests were completed by an Orion QuikRead go Instrument (Orion Corporation, Espoo, Finland); biochemical variables were completed by a Beckman AU5821 analyzer (Beckman Coulter, California, USA); and routine urinalysis was completed by Siemens Bayer Clinitek 500 analyzers (Siemens, Munich, Germany).

### Statistical analysis

R version 3.5.2 and Prism 8.2.0 were used to perform all the statistical analyses. Descriptive statistics were presented as the mean ± standard deviation for continuous data or frequencies for categorical variables. Student’s t test or one-way ANOVA was applied to test the mean differences between two groups or multiple groups with normal distributions, respectively; otherwise, the Wilcoxon rank sum test or Kruskal-Wallis test was performed.

The linkage analysis of the immunoglobulin proteome and all variables was performed by calculating the Pearson’s or Spearman’s correlation coefficient between two variables according to their normality. Pearson’s correlation coefficient was performed when the data of both variables had a normal or log-normal distribution. Hierarchical clustering analysis of the correlation coefficient matrices was performed using Euclidean distance and the complete method in the pheatmap package in R, with which the positively and negatively correlated variables were clustered together in a heatmap. A *P* value of < 0.05 was considered to be statistically significant.

In addition, ratios between every two clinical variables were calculated. Ratios more than one with significant difference when comparing BD-N and HC were retained for further comparison between groups in the validation set. A P value of < 0.05 was considered to be statistically significant.

## Results

### Quantification of the immunoglobulin proteome using a plasma microarray

**Fig. 1:**
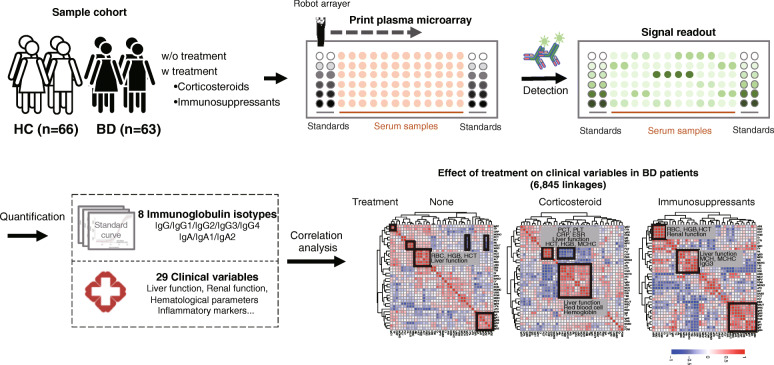
Flow chart of high-throughput immunoglobulin protein quantification by plasma microarray. Plasma samples from HCs and BD patients with or without corticosteroid and immunosuppressant treatment were printed on a microscope slide using an arrayer together with different concentrations of purified immunoglobulin standards. The detection of immunoglobulin in plasma was performed using fluorescein-labeled detection antibody, and the signal was read with a microarray fluorescent scanner at wavelengths of 532 nm and 635 nm, separately. The quantified immunoglobulin proteome was analyzed with 29 clinical variables for their correlations in BD patients without and with appropriate treatments. HC: healthy control; BD: Behcet’s disease

A schematic illustration of high-throughput immunoglobulin proteome detection in plasma and the following data analysis is shown in Fig. 1. Briefly, all 129 plasma samples were printed onto a microscope slide using a microarray together with a series of concentrations of immunoglobulin protein standards. The resulting array was then detected by a fluorescein-labeled anti-immunoglobulin secondary antibody within 30 min. Standard curves were constructed by using the signals from immunoglobulin protein standards, with which the concentrations of immunoglobulin protein in all plasma samples can be quantified (Fig. S1). Statistical and correlation analyses were employed to identify the variable linkages that are related to BD and clinical treatment with corticosteroids and immunosuppressants, respectively.

A representative image of the fluorescence detection of the plasma microarray is shown in Fig. S2A. The signal of the immunoglobulin IgA standard was increased with increasing concentrations of the protein standards. The IgA in plasma samples displayed different signals on the microarray. The r correlation within and between different arrays was 0.91 and 0.96, respectively (Fig. S2B). The r correlations within arrays are calculated between blocks for different samples in the same array. In addition, we printed identical proteins on the top and bottom of the slide to evaluate the effect of printing location on plasma protein detection. The results indicate that the r correlation between the two locations was 1.00 for the immunological proteome (IgA, IgA1–2, IgG, and IgG1–4) (Fig. S2C, D). All these results demonstrate the high reproducibility of immunoassays using plasma microarrays.

### Differential expression analysis of the plasma immunoglobulin proteome in BD patients receiving immunomodulatory therapy

Using this platform, we quantified the immunological proteome (IgA, IgA1–2, IgG, and IgG1–4) in the plasma of 66 HCs and 63 BD patients without and with corticosteroid and immunosuppressant treatment (Fig. 1). Prior to the statistical analysis, we analyzed the effect of age and sex on the expression of the immunoglobulin proteome. The results indicate that there was no correlation between immunoglobulin and age in either HCs or BD patients (Fig. S3A, B). The same results were obtained for sex and the immunoglobulin proteome in HCs (Fig. S4A). However, the expression of IgG4 in male BD patients was higher than that in female BD patients (*P* = 0.0326) (Fig. S4B).

**Fig. 2:**
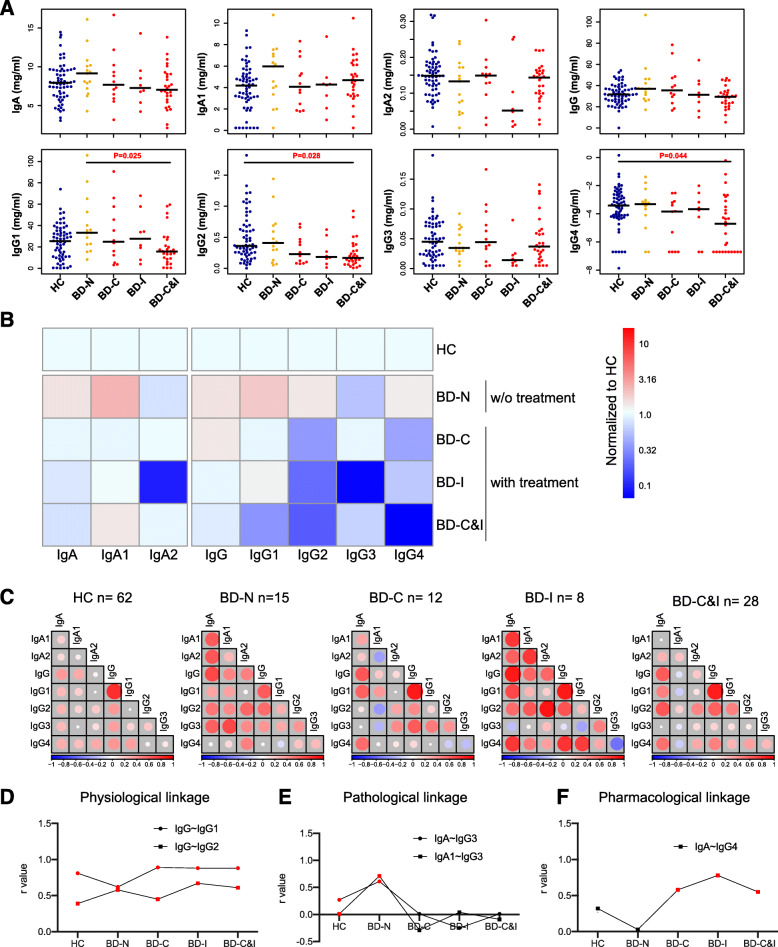
Analysis of the immunoglobulin proteome in the plasma of HCs and BD patients. **a**. Beeswarm plot of immunoglobulin proteome expression in HCs and BD patients with and without treatments; **b**. Comparison of immunoglobulin proteome changes in HCs and BD patients with and without treatments; **c**. Correlation analysis of immunoglobulin proteome in HC and BD patients with and without treatments; **d**-**f** are the changes in representative physiological, pathological and pharmacological linkages in HCs and BD patients with and without treatments, respectively. HC: healthy control; BD: Behcet’s disease

Compared to the HCs, no significant changes were observed in the expression levels of IgG, IgG1–4, IgA and IgA1–2 in patients with BD-N (Fig. 2a, b). The expression levels IgG1, IgG2 and IgG4 were suppressed by using combination treatment of corticosteroids and immunosuppressants (Fig. 2a, b) since statistical significance (*P* < 0.05) was achieved for IgG1 between BD patients without and with the combination of corticosteroids and immunosuppressant treatment, as well as for IgG2 and IgG4 between HCs and BD patients receiving the combination of corticosteroids and immunosuppressant treatment (P < 0.05, Fig. 2a). The results demonstrate that the immune system could be suppressed by corticosteroids and immunosuppressants through the regulation of immunoglobulin proteome expression.

Furthermore, we performed global linkage analysis to elucidate the potential mechanism promoted by the correlations of these immunological proteins during the progression of BD and the patients receiving immunomodulatory medication (Fig. 2c). We defined correlations (r) of 0.20–0.39 as weak linkage, 0.4–0.59 as moderate linkage, 0.60–0.79 as strong linkage and 0.8–1.0 as very strong linkage (Table S3). The red and blue colors of the circles represent positive and negative correlations, respectively. The size of the circle and intensity of the color are proportional to the correlation coefficients. We defined the physiological, pathological and pharmacological linkages according to the change of linkage under the disease and therapeutic situations. For example, the correlation of IgG-IgG1 (r = 0.62 ~ 0.89) and IgG-IgG2 (r = 0.39–0.67) remained constant in the HCs and BD patients without and with immunomodulatory therapy and were assigned as the physiological linkages (Fig. 2d). However, the correlations of IgA-IgG3 and IgA1-IgG3 were increased in BD patients compared with HCs and were assigned to the pharmacological linkage (Fig. 2e). Conversely, the correlation of pharmacological linkage, and IgA-IgG4 in the BD group was increased only in the patients who received immunomodulatory therapy (BD-C, BD-I and BD-C&I) (Fig. 2f).

### Analysis of clinical variables in BD patients received immunomodulatory therapy

**Fig. 3:**
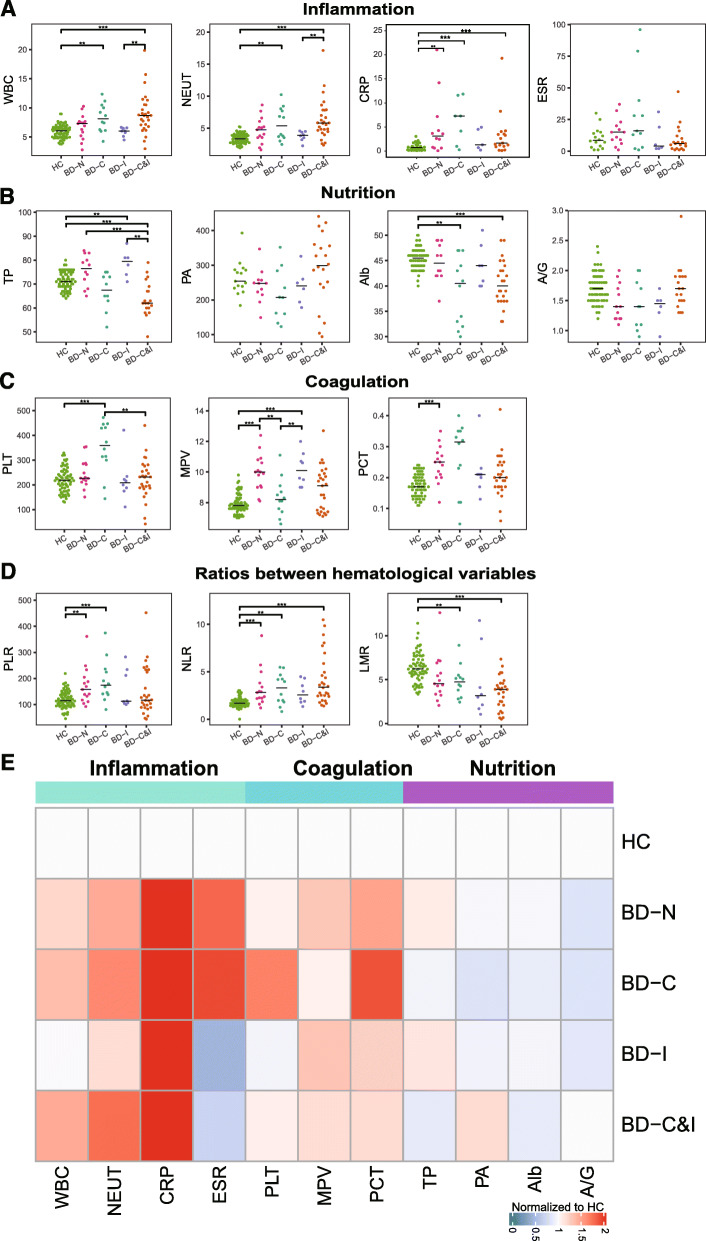
Changes in clinical variables in HC and BD patients. **a**-**d**. Beeswarm plot analysis of the changes in clinical variables associated with inflammation, coagulation, nutrition and the ratios of inflammatory cells in HCs and BD patients with and without treatments, respectively; **e**. Comparison of the changes in inflammation, coagulation, nutrition and the ratios of inflammatory cells in HCs and BD patients with and without treatments, respectively. HC: healthy control; BD: Behcet’s disease

We then analyzed the changes in 29 variables, which were associated with inflammation, coagulation and nutrition, from clinical tests in HCs and BD patients without and with therapeutic treatment (Fig. 3 and Fig. S5). The abbreviations of their full names are shown in Table S1.

First, we found that the clinical variables associated with inflammation are highly regulated in BD patients compared to HCs, including WBC, NEUT, CRP (Fig. 3a). The WBC level was high in BD patients treated with corticosteroids either alone or in combination with immunosuppressants (*P* < 0.05 for BD-C vs. HC). The same results were obtained for NEUT, CRP and ESR, which is in accordance with the function of immunosuppressants in inhibiting immunity and inflammation [8, 9, 21].In addition, compared to ESR, CRP was significantly elevated in the BD groups (*P* < 0.01), indicating that CRP is an effective indicator of inflammation in BD.

Second, in this study, we found no significant difference in nutrition variables between BD-N and HC (P < 0.05, Fig. 3b). However, the use of corticosteroids alone or in combination with the suppressants reduced the expression of albumin (Alb, *P* < 0.01 for BD-C vs. HC and BD-C&I vs. HC) and therefore downregulated the total protein levels, especially in the BD-C&I group (TP, *P* < 0.01 for BD-C&I vs. HC/BD-N/BD-I). Moreover, we found that Alb and TP decreased in patients with gastrointestinal involvement compared with those without gastrointestinal involvement and healthy controls, which may be due to impaired digestive function and a poor nutrition condition (Table S2).

Third, among these platelet variables associated with the coagulation process, MPV and PCT were observed to be significantly higher in the BD-N group than in the HC group (*P* < 0.01), indicating an increase in the volume of platelets and thus an enhanced ability of thrombosis [22]. Moreover, the use of corticosteroids increased the number of platelets (PLT, P < 0.01 for BD-C vs. HC and BD-C vs. BD-C&I) and induced a corresponding increase in PCT while decreasing MPV in BD patients by reducing the destruction of platelets (P < 0.01 for BD-C vs. BD-N and BD-C vs. BD-I) (Fig. 3c).

Fourth, we analyzed the changes in the ratio between these hematological indicators – neutrophil-to-lymphocyte ratio (NLR), platelet-to-lymphocyte ratio (PLR) and lymphocyte-to-monocyte ratio (LMR) in the HC and BD groups. NLR and PLR were increased in the BD groups compared with the HC group (P < 0.01), while LMR was decreased (P < 0.01) (Fig. 3d).

The heatmap analysis of these variables further demonstrated the increase in inflammation and coagulation as well as the decrease in nutritional status in BD patients without and with immunomodulatory treatment (Fig. 3e). All clinical variables with significant difference between BD-N and HC are listed in Table S2.

### Comprehensive linkage analysis of clinical variables in BD patients receiving immunomodulatory therapy

**Fig. 4:**
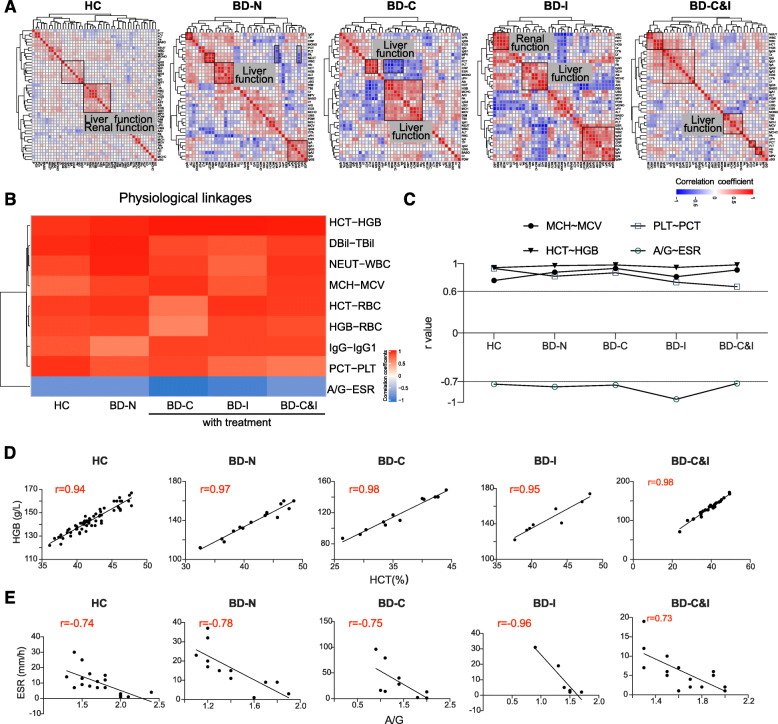
Comprehensive linkage analysis of all variables in BD patients receiving immunomodulatory therapy. **a**. Heatmap representing the correlation coefficient matrix for all 37 variables in HC and BD patients received with immunomodulatory therapy. Red represents a positive correlation, and blue represents a negative correlation. **b**. Heatmap of physiological linkages. **c**. The correlation changes in representative physiological linkages (MCH-MCV, HCT-HGB, PLT-PCT, and A/G- ESR) in HC and BD patients without and with therapy; **d**-**e**. The plot analysis of HCT-HGB and A/G-ESR correlations, respectively. HC: healthy control; BD: Behcet’s disease

Based on the previous finding, we speculated that all variables associated with our physiological system perform their functions cooperatively. To address this question, we comprehensively analyzed the linkages of all 37 variables in five groups of 129 samples by nonhierarchical clustering analysis, which led to 6845 linkages (Fig. 4a). The results indicate that the linkages of all variables were changed in the HCs and BD patients without and with immunomodulatory therapy, in which the linkages of the positive (red color) or negative (blue color) correlations were clustered and displayed as modules. For example, the variables of liver and renal functions were clustered together in the HC group, which is in accord with the collaborative function of the liver and kidneys to filter blood and process chemicals from food, medication and toxic substances. However, the kidneys could be damaged by BD, and some BD patients present with IgA nephropathy and amyloidosis [23, 24]. The results can be reflected in our correlation analysis in which the liver and renal function modules were separate in all BD groups (BD, BD-C, BD-I and BD-C&I).

Furthermore, the nonhierarchical clustering analyses revealed many physiological variable linkages that persisted in all HC and BD groups (Fig. 4b, Table 2). For example, a high positive correlation (r > 0.6) was observed for three physiological variable linkages (HCT-HGB, MCH- MCV and PLT- PCT) (Fig. 4c and d), confirming the functional association of these variables in oxygen transportation and coagulation [25, 26]. Conversely, A/G and ESR displayed a negative correlation in all HCs and BD patients (r = − 0.96 ~ − 0.73) (Fig. 4c and e), which confirmed their functional association with inflammation [27].

**Table 2:** Physiological, pathological and pharmacological linkages of clinical variables

Type	Linkage	HC	BD-N	BD-C	BD-I	BD-C&I
Pathological linkage	A/G-MCH	0.058	0.595	0.491	0.632	0.506
Pathological linkage	A/G-MCHC	0.053	0.499	0.779	0.783	0.339
Pathological linkage	A/G-PLT	− 0.059	− 0.215	− 0.771	− 0.667	− 0.176
Pathological linkage	Alb-DBil	0.037	0.116	0.424	0.818	0.192
Pathological linkage	Alb-IgG1	0.009	0.42	0.049	0.055	0.335
Pathological linkage	Alb-uSG	0.042	0.834	0.584	0.783	0.439
Pathological linkage	Cr-upH	−0.092	− 0.377	− 0.25	− 0.447	− 0.426
Pathological linkage	HGB-TP	0.104	0.256	0.281	0.812	0.345
Pathological linkage	IgA2-IgG	0.051	0.493	0.147	0.595	0.209
Pathological linkage	IgG-LY%	0.053	0.157	0.28	0.333	0.333
Pathological linkage	IgG1-IgG2	0.009	0.304	0.168	0.262	0.481
Pathological linkage	LY%-WBC	−0.05	− 0.7	− 0.636	− 0.452	− 0.266
Pathological linkage	MCH-MONO	− 0.061	− 0.266	− 0.671	−0.156	− 0.5
Pathological linkage	MCH-PLT	−0.069	−0.181	− 0.504	−0.743	− 0.196
Pathological linkage	MCHC-PLT	−0.042	−0.215	− 0.634	−0.167	− 0.104
Pathological linkage	MCV-PLT	−0.056	−0.218	− 0.601	−0.539	− 0.22
Pathological linkage	PDW-TP	−0.006	−0.35	− 0.049	−0.493	− 0.592
Pathological linkage	BASO-Cr	−0.021	0.365	0.172	0.11	0.37
Pathological linkage	BASO-LY%	−0.003	0.246	0.244	0.085	0.248
Pathological linkage	IgG-MCHC	0.011	−0.293	− 0.445	−0.381	− 0.329
Pathological linkage	IgG2-LY	−0.074	0.379	0.49	0.262	0.487
Pathological linkage	MCHC-MONO	0.077	−0.264	−0.473	− 0.643	−0.613
Pathological linkage	upH-TP	0.049	−0.64	− 0.682	− 0.816	− 0.12
Pathological linkage	uSG-TP	− 0.184	0.781	0.463	0.816	0.439
Pharmacological linkage	A/G-PA	0.1286	0.028	0.685	0.377	0.458
Pharmacological linkage	A/G-uSG	0.182	0.024	0.425	0.258	0.067
Pharmacological linkage	EOS-IgG4	−0.009	−0.014	−0.063	− 0.321	− 0.27
Pharmacological linkage	HCT-IgG4	0.244	−0.015	−0.334	− 0.436	−0.109
Pharmacological linkage	IgA-IgG4	−0.4763	−0.032	− 0.153	−0.986	− 0.337
Pharmacological linkage	IgG-IgG4	0.026	0.007	0.307	0.067	0.271
Pharmacological linkage	IgG-LY	0.1786	−0.14	−0.8	−0.551	− 0.394
Pharmacological linkage	MCHC-PA	−0.4639	−0.027	− 0.338	−0.899	− 0.524
Pharmacological linkage	A/G-DBil	−0.05007	0.014	0.041	0.841	0.056
Pharmacological linkage	A/G-TBil	0.056	0.009	0.402	0.036	0.539
Pharmacological linkage	CRP-PLT	0.364	0.032	0.577	0.778	0.518
Pharmacological linkage	HGB-MCHC	0.418	0.02	0.149	0.79	0.488
Pharmacological linkage	IgG4-TP	0.079	0.093	0.319	0.619	0.444
Pharmacological linkage	PDW-TBil	0.143	−0.002	−0.487	−0.143	−0.033
Pharmacological linkage	ESR-WBC	0.2504	0.088	0.632	0.771	0.351
Pharmacological linkage	IgA-NEUT	−0.178	−0.028	−0.6	− 0.108	−0.304
Pharmacological linkage	BASO-NEUT	0.113	−0.022	0.36	0.986	0.065
Pharmacological linkage	ESR-PA	0.143	−0.042	0.35	0.986	0.341
Pharmacological linkage	IgG2-MCHC	0.07099	0.06	−0.61	−0.464	− 0.589
Pharmacological linkage	Alb-CRP	0.2121	0.005	−0.874	−0.429	− 0.697
Pharmacological linkage	CRP-PA	0.04547	−0.039	0.796	0.314	0.269
Pharmacological linkage	IgG-upH	−0.1039	0.077	−0.18	−0.486	− 0.176
Pharmacological linkage	IgG4-upH	−0.118	0.063	−0.216	−0.335	− 0.415
Pharmacological linkage	MCHC-WBC	−0.2585	0.005	−0.323	−0.272	− 0.237
Pharmacological linkage	Alb-PDW	0.276	−0.027	0.616	0.69	0.218
Pharmacological linkage	DBil-ESR	−0.013	−0.107	0.259	0.333	0.264
Pharmacological linkage	ESR-TBil	0.118	0.075	−0.241	− 0.671	−0.256
Pharmacological linkage	MCV-MONO	0.124	0.006	−0.633	−0.335	− 0.236
Pharmacological linkage	EOS-PA	0.176	−0.095	0.508	0.551	0.331
Pharmacological linkage	EOS-upH	0.036	0.005	−0.021	−0.476	− 0.335
Pharmacological linkage	ESR-uSG	0.188	−0.021	0.459	0.324	0.047
Pharmacological linkage	BASO-LY	0.351	0.439	0.027	0.073	0.216
Pharmacological linkage	CRP-MONO	0.4561	0.599	0.116	0.086	0.275
Pharmacological linkage	BASO-ESR	−0.159	−0.332	−0.014	−0.024	− 0.066
Pharmacological linkage	IgG4-NEUT	−0.074	0.576	−0.034	−0.044	− 0.027
Pharmacological linkage	EOS-IgA	0.2716	−0.631	0.04	0.206	0.042
Pharmacological linkage	LY%-PLT	−0.012	−0.617	0.065	0.108	0.253
Pharmacological linkage	A/G-BASO	0.067	−0.624	0.203	0.252	0.107
Physiological linkage	DBil-TBil	0.949	0.968	0.859	0.821	0.9
Physiological linkage	HCT-HGB	0.928	0.957	0.977	0.976	0.97
Physiological linkage	HCT-RBC	0.898	0.928	0.685	0.929	0.904
Physiological linkage	HGB-RBC	0.861	0.879	0.609	0.881	0.844
Physiological linkage	IgG-IgG1	0.814	0.621	0.888	0.881	0.878
Physiological linkage	MCH-MCV	0.752	0.877	0.932	0.813	0.909
Physiological linkage	NEUT-WBC	0.863	0.961	0.895	0.762	0.925
Physiological linkage	PCT-PLT	0.933	0.819	0.868	0.732	0.668
Physiological linkage	A/G-ESR	−0.754	−0.751	−0.956	−0.896	−0.74

**Fig. 5:**
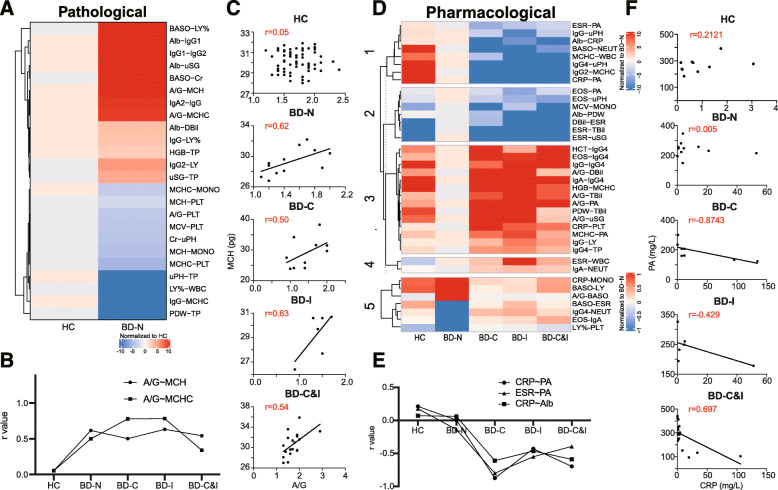
Comprehensive analysis of pathological and pharmacological linkages in BD patients receiving immunomodulatory therapy. **a**. Heatmap of pathological linkages; **b**. The correlation changes in representative pathological linkages (A/G-MCH and A/G-MCHC) in HCs and BD patients without and with immunomodulatory therapy; **c**. The plot analysis of A/G-MCH correlations in HC and BD patients without and with immunomodulatory therapy; **d**. Heatmap of pharmacological linkages; **e**. The correlation changes in representative pharmacological linkages (CRP-PA, ESR-PA and CRP-Alb) in HCs and BD patients without and with immunomodulatory therapy; **f**. The plot analysis of CRP-PA correlations in HCs and BD patients without and with immunomodulatory therapy. HC: healthy control; BD: Behcet’s disease

In addition, we noticed pathological and pharmacological variable linkages that were shown in BD patients without and with immunomodulatory treatment compared to the HCs (Table 2). For example, four linkages (A/G-MCH, A/G-MCHC, Alb-uSG, TP-uSG) were changed from none to positive correlations (r = 0.339–0.834) in the BD patients regardless of treatment (Fig. 5a-c), indicating the role of nutrition in BD pathogeneses [28–30]. As important inflammatory and thrombosis markers in BD, WBC (r = 0.532, *P* = 0.04) and NEUT (r = 0.593, *P* = 0.02) showed a significantly positively correlation with PLT in BD patients without immunomodulatory treatment (Fig. 4, data not shown), suggesting their role of interactions in chronic infection, inflammation and tissue lesions in BD.

The pharmacological linkages were clustered into five modules in BD patients under treatment (Fig. 5d). The correlation of linkages in the BD group was lower in modules #1-#4 and higher in module #5 than in the other groups. Compared to the HC group, the negative correlation of pharmacological linkages in module #2 was decreased in BD patients and then increased in patients with treatments. The same results were observed in module #3 with positive correlations in HCs and treated patients. The linkages in module #1 changed from positive correlations in the HC group to negative correlations in treated groups, which is in contrast to module #4. Notably, the inflammation variables (CRP and ESR) showed no correlation with the nutritional variables (Alb and PA) in BD patients. However, their linkages were changed to positive correlations in BD patients after treatment with corticosteroids or immunosuppressants (Fig. 5d-f), which is in accord with previous reports [31, 32] and confirms the association between the increase in inflammation and poor nutrition. However, the clinical utility of these pharmacological linkages has to be validated in different cohorts of BD patients with the follow-up information.

### Analysis of ratio changes between clinical variables in patients with BD and healthy and disease controls

Based on the linkage analysis, we further calculated the ratios between every two clinical variables to investigate their coordinated changes. In total, there were 152 pairs of variables with significant fold changes of more than one between BD-N and HC (Table S4).

**Fig. 6:**
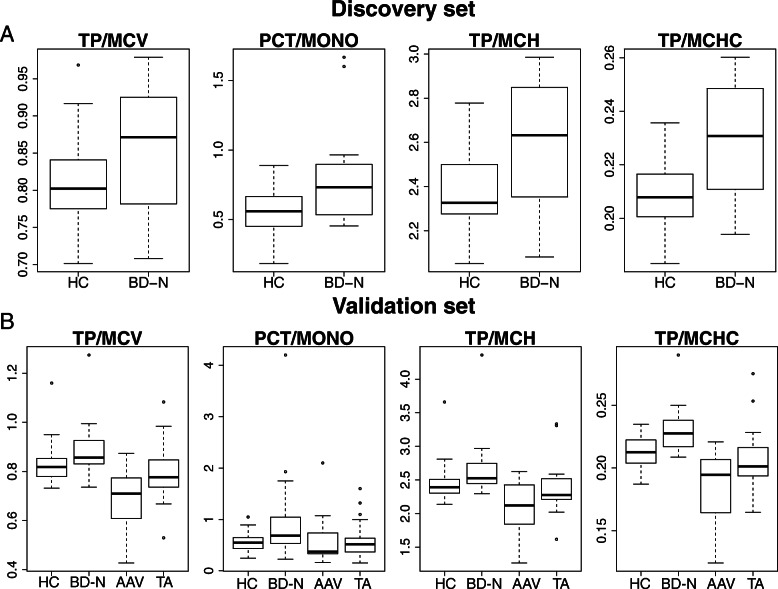
Significant ratio changes between BD and HC and disease controls in the discovery and validation sets. **a**. Boxplot of the levels of the four ratios in HCs and BD patients with and without treatments in the discovery set. **b**. Boxplot of the levels of the four ratios in HCs, BD patients with and without treatments, and patients with ANCA-associated vasculitis and Takayasu arteritis in the validation set. HC: healthy controls; BD-N: BD patients not receiving corticosteroid or immunosuppressant treatment; TA: Takayasu arteritis; AAV: ANCA-associated vasculitis

To validate the significant changes between two clinical variables, the immunoglobulin expression and clinical variables in a validation set consisting of BD-N (*n* = 27), BD-C (*n* = 5), BD-I (*n* = 16), HC (*n* = 30), AAV (*n* = 18), and TA (n = 18) (Table 1) were measured. Significant ratio changes for 8 pairs of variables were ultimately validated in the discovery set (Table S5), among which TP/MCV, PCT/MONO, TP/MCH, and TP/MCHC were found to be significantly increased in BD compared with HC, AAV and TA in the discovery and validation sets (*P* < 0.05, Fig. 6, Table 3; Table S5), regardless of immunomodulatory therapy (*P* > 0.05, Table 3; Table S5). In addition, these biomarkers were not affected by corticosteroids and immunosuppressants (P > 0.05 for BD-N vs. BD-C and P > 0.05 for BD-N vs. BD-I, Table S5).

**Table 3:** Ratios significantly increased in BD patients without corticosteroid and immunosuppressant therapy compared with healthy controls and disease controls in both the discovery and validation sets

	Discovery set BD-N vs.		Validation set: BD-N vs.					
	HC		HC		AAV		TA	
	p	fc	p	fc	p	fc	p	fc
**TP/MCV**	*	1.0579355	*	1.0712601	***	1.2863344	*	1.120872
**PCT/MONO**	**	1.425393	*	1.5778604	*	1.6435788	*	1.5614405
**TP/MCH**	**	1.0904052	*	1.1113632	***	1.2590039	*	1.0998654
**TP/MCHC**	**	1.0932365	**	1.1448527	***	1.2285696	*	1.091615

* *P* < 0.05. ** *P* < 0.01. *** *P* < 0.001

Furthermore, we investigated changes in the ratio of different BD subsets and found that due to the decreased TP levels, TP/MCV, TP/MCH and TP/MCHC were decreased in BD patients with gastrointestinal involvement compared with those without gastrointestinal involvement and healthy controls. Conversely, significant increases in TP/MCV, TP/MCH and TP/MCHC were found in BD patients without gastrointestinal involvement compared to healthy controls. MPV/HCT and MPV/HGB were also increased in BD patients (Table S5), especially the BD subset with blood system involvement, including five patients with anemia or myelodysplastic syndrome (Table S5), indicating anemia and potential disorder of blood cell morphology.

## Discussion

BD is an inflammatory disease of unknown etiology that affects the epidermal, mucocutaneous, vascular, ophthalmologic, gastrointestinal, pulmonary, and central nervous systems. Corticosteroids and immunosuppressants are frequently employed clinically to treat BD patients by regulating inflammation and immune disorders. As an option for long-term treatment for autoimmune diseases [33], immunosuppressants have an inhibitory effect on the immune response to weaken attacks on body’s own tissue by inhibiting the proliferation and function of T cells or B cells [34]. In contrast, corticosteroids work quickly but have significant side effects. Corticosteroids can affect almost all kinds of immune cells and multiple points of the immune response. For example, they prevent lymphocyte recycling and the production of antibody-producing and cytotoxic effector cells, but they also have significant anti-inflammatory effects. They inhibit the adhesion of neutrophils to vascular endothelium in inflammatory sites and inhibit monocyte function, among other effects [35]. However, the pathogenesis of BD and its therapeutic influence by immunomodulatory medication are largely unknown. To address this question, we comprehensively measured and analyzed the changes in clinical variables related to immunity, inflammation, coagulation and nutrition in HCs and BD patients without and with immunomodulatory medication.

We observed an overall increase in immunoglobulin proteome expression in BD patients without treatment (Fig. 2), which demonstrates the existence of an immune disorder during BD development [4]. However, the expression of the immunoglobulin proteome, especially IgG1, IgG2 and IgG4, can be suppressed by corticosteroids and immunosuppressants. The results can be further confirmed by the correlation analysis, in which the correlations of pathological linkages (IgA-IgG3 and IgA1- IgG3) were increased in the BD group and decreased under immunomodulatory therapy (Figs. 2c and 3e). The results demonstrate that corticosteroids and immunosuppressants exert their effects by inhibiting immune and inflammatory responses [4, 7].

The same results were observed in inflammation, in which WBC and NEUT were significantly increased in the BD groups, which was consistent with the functions of WBC and NEUT in mediating vessel damage through enhanced migration in the circulatory system. While corticosteroids are used to inhibit inflammation and the immune response in certain clinical situations, they may also cause an increase in the WBC count and predominantly neutrophils (NEUT) mainly by the demargination of the neutrophils from the endovascular lining [36, 37]. In addition, the use of corticosteroids may promote the maturation of neutrophils in the bone marrow and mobilization into the blood circulation by expression of key receptors such as Annexin A1 [38, 39], as also observed for the pathological link of BASO-LY% and LY%-WBC. Although immunosuppressants such as azathioprine are reported to causes dose-related bone marrow suppression and leukopenia [40], we did not observe significant difference in blood cells in this study. All these results suggest that clinical evaluations of inflammation should consider medication use as well as the clinical symptoms and signs of the patients.

Inflammation may cause damage to the vessel wall and initiate the coagulation pathway and thrombosis [22, 41]. Platelets could be hyperactivated under inflammation, after which granules are released to further promote coagulation and inflammation [42]. In this study, upregulation of MPV in BD patients and its downregulation by corticosteroids were observed (Fig. 3). MPV reflects alterations in the morphology of platelets. Elevated MPV means larger platelets with more dense granules that are therefore more thrombogenic than smaller ones, and it is a marker of platelet function and is involved in thrombosis and vascular damage in BD [43, 44]. In contrast, the change in PLT was not obvious, as changes in MPV and PCT can be observed before detectable changes in platelets [22]. There is also growing evidence that platelets are not only involved in fatal vascular events but also function in disease progression by interacting with neutrophils. Schrottmaier et al. proposed that direct interaction of platelets with neutrophils leads to neutrophil activation, recruitment and formation of neutrophil extracellular traps, further promoting the progression of vascular pathologies [42]. Pamuk et al. found significantly higher levels of platelet-neutrophil complexes in BD patients with major vascular involvement than in those without vascular involvement and healthy controls [45]. Consistently, interaction between WBCs or neutrophils and platelet was observed in this research in BD patients based on their positive correlation (Fig. 4). Platelets also showed the highest affinity for other innate and adaptive immune cells, including monocytes (as discussed in the next section) and lymphocytes, by soluble mediators [42]. In this study, the pharmacological linkages of LY%-PLT and CRP-PLT further suggest that integrative analysis of granulocytes, platelets and related variables is likely to provide a comprehensive understanding of disease activity, thrombotic potential and potential tissue damage.

Based on linkage analysis, we further constructed a novel method according to the ratio changes between two clinical variables and demonstrated that four ratios – TP/MCV, PCT/MONO, TP/MCH, and TP/MCHC – have higher value in BD than in HC, TA and AAV, suggesting these four ratios as potential diagnostic indicators for BD. PCT, which is produced from PLT and MPV, reflects the total platelet mass [46]. In our research, PCT and MPV were significantly increased in BD. Platelets play an important role in the pathogenesis of thromboembolic diseases. Platelets are more reactive in BD patients than in normal controls, which may contribute to the tendency for thrombosis. Moreover, increased MPV in an inflammatory state contributes to thrombosis, which may be an independent risk factor for vascular involvement in BD [47]. Evidence has shown that monocytes in BD patients are activated and produce proinflammatory cytokines, causing increased adhesion of neutrophils to endothelial cells and chronic inflammation [48]. Interactions between platelets and monocytes is also reported to relate to major vascular involvement in BD [45], and platelets may induce monocyte differentiation into a more inflammatory phenotype [49]. The higher value of PCT/MONO, consisting of platelets and monocytes, confirms the potentially close interaction between platelets and monocytes [42]. This has been highlighted as an important pathophysiological link between inflammation, thrombosis and endothelial activation [50], such as the concordance of platelets and monocytes in immune-thrombosis. Moreover, platelets are reported to interact with monocytes to propagate their differentiation into macrophages, and when activated, platelets stimulate monocytes to leave the blood vessel and enter tissues, causing a higher level of PCT/MONO [51]. We propose that a higher level of PCT/MONO, representing aggregates and interaction between platelet and monocyte, is a potentially attractive and easily accessible marker in BD [42].

MCV, MCH, and MCHC are useful biomarkers in the evaluation of anemia. MCV indicates the mean size of red blood cells, while MCH and MCHC indicate the mean amount and the mean concentration of hemoglobin in each red blood cell, respectively. It has been reported that chronic anemia is common in BD patients, especially with intestinal involvement [52, 53], with contributors like bone marrow failure [54] or serum prohepcidin and hepcidin, whose levels are also closely associated with disease activity [55]. It is likely that the increasing trend of total protein and/or decreases in MCV, MCH or MCHC lead to high levels for the three ratios. However, our study demonstrated that corticosteroids and immunosuppressants do not function by decreasing these higher ratios, illustrating the stability of these indicators. However, other factors involved and the specific mechanisms of these interactions remain to be elucidated in future studies.

There are several limitations in our research. First, the coregulatory mechanisms of clinical variables through physiological, pathological and pharmacological linkages are not well understood and should be carefully interpreted according to the clinical symptoms of BD patients. Second, the numbers of samples and patients’ information employed in this study were limited. In the future, we will include more information to match the backgrounds of control patients and verify the utility of these functional linkages in diagnosis and prognosis in larger cohorts.

## Conclusion

In this work, we measured and performed a comprehensive correlation analysis of clinical variables for BD patients with appropriate therapeutic treatment. It is important for clinicians to be aware of the effect of immunomodulatory therapy on laboratory tests for an appropriate interpretation of patients’ conditions and to reduce unnecessary medical examinations or therapies. Moreover, we conceptually defined the physiological, pathological and pharmacological linkages of these variables and elucidated their functions for clinical application. The linkages found in our study highlight the close interactions between several markers in BD. Mutual regulation of platelets and different immune cells or other inflammatory markers promotes the development and exacerbation of vascular abnormalities in the pathogenesis of BD, suggesting the potential of combined anti-coagulation and anti-inflammation therapy in BD. The ratio changes among PLT/MONO, TP/MCV, TP/MCH and TP/MCHC are stable diagnostic indicators for BD regardless of the medication status. These results demonstrate the utility of our approach in elucidating the potential mechanism of BD pathogenesis and therapeutic effects as well as in identifying potential biomarkers to assist BD diagnosis and therapies in the future.

## Supplementary Information

**Additional file 1: Table S1** Abbreviations and full names of the laboratory tests used in this study. **Table S2** Variables with significant differences between healthy controls and BD patients not treated with corticosteroids and immunosuppressants therapy. **Table S3** Spearman correlation (r and *P* value) analysis of the immunoglobulin proteome in HCs and BD patients with and without immunomodulatory therapy. **Table S4** Ratios with significant differences and fold change more than one between healthy controls and BD patients not treated with corticosteroids and immunosuppressants therapy in the discovery set. **Table S5** Ratios with significant differences between BD patients not treated with corticosteroids and immunosuppressants therapy, healthy control and disease controls in both the discovery set and validation set. **Fig. S1.** Standard curves for the quantification of eight immunoglobulin isotypes. **Fig. S2.** The assay performance of the plasma microarray in the detection of the immunoglobulin proteome. **Fig. S3.** Correlation analysis between immunoglobulins and age in HC(A) and BD(B). **Fig. S4.** Beeswarm plots of immunoglobulin proteome expression between sex groups in HC(A) and BD(B). **Fig. S5.** Comparison of laboratory tests in HC and BD patients with and without immunomodulatory therapy.

## Data Availability

All data generated or analyzed during this study are included in this published article [and its supplementary information files].
