# Supplementary material for: Comprehensive analysis of immunoglobulin and clinical variables identifies functional linkages and diagnostic indicators associated with Behcet’s disease patients receiving immunomodulatory treatment
Source: BMC Immunol. 2021 Feb 22;22:16. doi: 10.1186/s12865-021-00403-1 (PMC7901184; doi:10.1186/s12865-021-00403-1)
Supplement: Supplementary file 1 — Additional file 1: Table S1 Abbreviations and full names of the laboratory tests used in this study. Table S2 Variables with significant differences between healthy controls and BD patients not treated with corticosteroids and immunosuppressants therapy. Table S3 Spearman correlation (r and P value) analysis of the immunoglobulin proteome in HCs and BD patients with and without immunomodulatory therapy. Table S4 Ratios with significant differences and fold change more than one between healthy controls and BD patients not treated with corticosteroids and immunosuppressants therapy in the discovery set. Table S5 Ratios with significant differences between BD patients not treated with corticosteroids and immunosuppressants therapy, healthy control and disease controls in both the discovery set and validation set. Fig. S1. Standard curves for the quantification of eight immunoglobulin isotypes. Fig. S2. The assay performance of the plasma microarray in the detection of the immunoglobulin proteome. Fig. S3. Correlation analysis between immunoglobulins and age in HC(A) and BD(B). Fig. S4. Beeswarm plots of immunoglobulin proteome expression between sex groups in HC(A) and BD(B). Fig. S5. Comparison of laboratory tests in HC and BD patients with and without immunomodulatory therapy. [file 12865_2021_403_MOESM1_ESM.zip › Revised supplementary data.docx]

**Comprehensive analysis of immunoglobulin and clinical variables identifies functional linkages and diagnostic indicators associated with Behcet’s disease patients receiving immunomodulatory treatment**

Linlin Cheng^a^, Yang Li^b^, Ziyan Wu^c^, Liubing Li^a^, Chenxi Liu^a^, Jianhua Liu^d^, Jiayu Dai^b^, Wenjie Zheng^c^, Fengchun Zhang^c^, Xiaobo Yu^b*^, Yongzhe Li^a*^

**Author unit:**

a 100730Department of Clinical Laboratory, Peking Union Medical College Hospital, Peking Union Medical College and Chinese Academy of Medical Sciences, Beijing, China.

b 102206State Key Laboratory of Proteomics, Beijing Proteome Research Center, National Center for Protein Sciences, Beijing Institute of Lifeomics, Beijing, China.

c 100730Department of Rheumatology and Clinical Immunology, Peking Union Medical College Hospital, Peking Union Medical College and Chinese Academy of Medical Sciences, Key Laboratory of Rheumatology and Clinical Immunology, Ministry of Education, Beijing, China.

d 110004Department of Laboratory Medicine, Shengjing Hospital of China Medical University, Shenyang, China.

***Corresponding author:**

Yongzhe Li. (yongzhelipumch@126.com; Tel: 86 (10)69159716); Xiaobo Yu. (yuxiaobo@mail.ncpsb.org; Tel: 86 (10)61777093).

Supplementary Tables: 5

Supplemental Figures: 5

**Supplemental Tables**

Table S2 Variables with significant differences between healthy controls and BD patients not treated with corticosteroids and immunosuppressants therapy.

Table S3 Spearman correlation (r and P value) analysis of the immunoglobulin proteome in HCs and BD patients with and without immunomodulatory therapy.

Table S4 Ratios with significant differences and fold change more than one between healthy controls and BD patients not treated with corticosteroids and immunosuppressants therapy in the discovery set.

Table S5 Ratios with significant differences between BD patients not treated with corticosteroids and immunosuppressants therapy, healthy control and disease controls in both the discovery set and validation set.

**Supplemental Figures**

| Figure S1 | Standard curves for the quantification of eight immunoglobulin isotypes. |
| --- | --- |
| Figure S2 | The assay performance of the plasma microarray in the detection of the immunoglobulin proteome. |
| Figure S3 | Correlation analysis between immunoglobulins and age in HC(A) and BD(B). |
| Figure S4 | Beeswarm plots of immunoglobulin proteome expression between sex groups in HC(A) and BD(B). |
| Figure S5 | Comparison of laboratory tests in HC and BD patients with and without immunomodulatory therapy. |

**Table S1 Abbreviations and full names of the laboratory tests used in this study.**

| **Abbreviation** | **Full name** | **Interpretation** |
| --- | --- | --- |
| A/G | Albumin to globulin ratio | Liver function: nutrition |
| Alb | Albumin | Liver function: nutrition |
| ALT | Alanine aminotransferase | Liver function: liver enzyme |
| ALP | Alkaline phosphatase | Liver function: liver enzyme |
| AST | Aspartate aminotransferase | Liver function: liver enzyme |
| BASO | Basophil | Hematological variables: basophil |
| Ca | Calcium | Electrolyte |
| Cl | Chlorine | Electrolyte |
| Cr | Creatinine | Renal function |
| CRP | C-reactive protein | Inflammation |
| DBil | Direct bilirubin | Liver function: bilirubin |
| EOS | Eosinophil | Hematological variables: eosinophil |
| ESR | Erythrocyte sedimentation rate | Inflammation |
| GGT | Gama glutamyl transferase | Liver function: Liver enzyme |
| Glu | Glucose | Glucose in the blood: energy source |
| HCT | Hematocrit | Hematological variables: red blood cell |
| HGB | Hemoglobin | Hematological variables: hemoglobin |
| K | Potassium | Electrolyte |
| LD | Lactate dehydrogenase | Liver function: Liver enzyme |
| LMR | lymphocyte to monocyte ratio | Ratios of hematological variables |
| LY | Lymphocyte | Hematological variables: lymphocyte |
| LY% | Lymphocytes percentage | Hematological variables: lymphocyte |
| MCH | Mean corpuscular hemoglobin | Hematological variables: hemoglobin |
| MCHC | Mean corpuscular hemoglobin concentration | Hematological variables: hemoglobin |
| MCV | Mean corpuscular volume | Hematological variables: red blood cell |
| MONO | Monocyte | Hematological variables: monocyte |
| MPV | Mean platelet volume | Hematological variables: platelet |
| Na | Sodium | Electrolyte |
| NEUT | Neutrophil | Hematological variables: neutrophil |
| NLR | Neutrophil to lymphocyte ratio | Ratios of hematological variables |
| P | Phosphorus | Electrolyte |
| PA | Prealbumin | Liver function: Nutrition |
| PCT | Plateletcrit | Hematological variables: platelet |
| PLR | Platelet to lymphocyte ratio | Ratios of hematological variables |
| PLT | Platelet | Hematological variables: platelet |
| RBC | Red blood cell | Hematological variables: red blood cell |
| TBil | Total bilirubin | Liver function: bilirubin |
| TP | Total protein | Liver function: Nutrition |
| UA | Uric acid | Urine tests |
| UBG | Urobilinogen | Urine tests |
| upH | Urine pH | Urine tests |
| uSG | Urine specific gravity | Urine tests |
| WBC | White blood cell | Hematological variables: white blood cell |

**Table S2 Variables with significant differences between healthy controls and BD patients not treated with corticosteroids and immunosuppressants therapy.**

**See Supplementary Table S2.xlsx**

**Table S3 Spearman correlation (r and p value) analysis of the immunoglobulin proteome in HCs and BD patients with and without immunomodulatory therapy.**

**See Supplementary Table S3.xlsx**

**Table S4 Ratios with significant differences and fold change more than one between healthy controls and BD patients not treated with corticosteroids and immunosuppressants therapy in the discovery set.**

**See Supplementary Table S4.xlsx**

**Table S5 Ratios with significant differences between BD patients, healthy control and disease controls in both the discovery set and validation set.**

**See Supplementary Table S5.xlsx**

**Figure S1 Standard curves for the quantification of eight immunoglobulin isotypes.** Standard curves were established after the signal intensity of the nine protein standards was fitted with a 4- or 5-parameter logistic model. The choice of the 4- or 5-parameter model depended on the goodness of fit of an R-squared regression. Absolute quantification of plasma immunoglobulin concentrations was performed through standard curves.

**Figure S2 The assay performance of plasma microarray in the detection of the immunoglobulin proteome.** A. Representative fluorescence image for IgA detection. B. Intra- and inter-variations of immunoglobulin protein detection using a plasma microarray. The intra- and intervariations were calculated using the data within the array (between blocks for detection of different samples) and between different arrays, respectively. C. Printing of different concentrations of immunoglobulin proteins at the top and bottom of the slide. D. Influence of array location on protein detection using plasma microarray. The r correlation of two identical arrays at the top and bottom of the slide was calculated.

**Figure S3 Correlation analysis between immunoglobulin and age in HC(A) and BD(B).** HC: healthy control; BD: Behcet’s disease.

**Figure S4 Beeswarm plots of immunoglobulin proteome expression between sex groups in HC(A) and BD(B).** HC: healthy control; BD: Behcet’s disease.

**Figure S5 Comparison of laboratory tests in HC and BD patients with and without immunomodulatory therapy.** These laboratory indicators are divided into three groups based on their relationship with red blood cells (A), other cell types (B) and liver and renal function (C). (D) Heatmap analysis of variables changes in HC and BD patients with and without immunomodulatory therapy. HC: healthy control; BD: Behcet’s disease.
